# Low-Cost Fishhook Removal Simulation

**DOI:** 10.21980/J8Q64P

**Published:** 2023-10-31

**Authors:** David Mitchell Baskin, Christopher Ashby Davis

**Affiliations:** * Wake Forest University School of Medicine, Department of Emergency Medicine, Winston-Salem, NC

## Abstract

**Audience:**

The target audiences for this hands-on innovation are health care providers including medical students and emergency medicine residents. This simulation is also appropriate for small group sessions teaching the layperson.

**Background:**

While generally not life-threating fishhook injuries are commonplace. They can end a day of recreation or an outdoor trip and possibly result in a visit to an emergency department or urgent care. Hands-on education on fishhook removal techniques that minimize tissue damage is rarely provided in wilderness first aid or traditional medical education. To the best of our knowledge, to date there are only two studies on fishhook removal simulations in medical and wilderness first aid education.^1^,^2^ The previously described simulation models are limited by accessibility of materials, realism, and cost.

**Educational Objectives:**

The goal of this small group session is to fill the gap in training on fishhook injuries. At the end of the session participants should be able to describe the parts of a fishhook, as well as demonstrate and have increased confidence in performing multiple fishhook removal techniques.

**Educational Methods:**

Social learning theory is the conceptual framework for this small group session.^3^,^4^ This reflects the idea that students learn not only through repetition with trial and error, but through social interactions, observing and modeling successes of others. As a result, while this simulation requires a facilitator ensure the required items are available it does not necessitate a facilitator be present over the entire duration. Participants perform common fishhook removal techniques with hands-on skill development using commercially available silicone sponge injection pad trainers.

**Research Methods:**

Evaluating this small group session at a wilderness medicine training attended by medical and physician assistant students and their guests, self-reported confidence in fishhook removal before and after the simulation was assessed with a paired t-test. Survey results of perceived effectiveness and value of the simulation were also evaluated.

**Results:**

The average confidence increased 58% after the simulation (p<0.005). The mean level of effectiveness was 87% and the participant perceived monetary value of the simulation materials was greater than actual cost.

**Discussion:**

This innovation is a cost-friendly way to provide education and practice on fishhook removal. It requires minimal set up time and pre-learning can be easily modified to the expected knowledge and experience of participants. Understanding the fishhook removal techniques and increased levels of confidence has the potential to make participants more efficient when caring for patients. It may result in greater likelihood of success in removing fishhooks with minimal tissue damage.

**Topics:**

Fishhook injuries, medical simulation, emergency medical education, wilderness first aid, wound management, injection pad trainers.

## USER GUIDE

[Table t1-jetem-8-4-i1]

**Table t1-jetem-8-4-i1:** 

List of Resources:	
Abstract	1
User Guide	3
Learner Materials	8
Instructor Materials	11

**Learner Audience:**

Medical Students, Interns, Junior Residents, Senior Residents

**Time Required for Implementation:**

30–40 minutes

**Recommended Number of Learners per Instructor:**

4–6

**Topics:**

Fishhook injuries, medical simulation, emergency medical education, wilderness first aid, wound management, injection pad trainers.

**Objectives:**

By the end of the small group session participants should be able to:

1. Recall basic principles of wound management and indications for tetanus prophylaxis.
2. Verbalize fishhook terminology and describe the steps of different fishhook removal techniques.
3. Determine an appropriate fishhook removal method and safely demonstrate its use with proper patient positioning and minimal tissue damage.

### Linked objectives and methods

Self-directed and hands-on learning using charts and diagrams, and commercially available silicon sponge injection pad trainers that replicate human skin, make this small group session engaging and allows participants to effectively meet the session objectives.

A chart on tetanus prophylaxis and fishhook diagram prepared for the session allows participants to actively work together (objective 1). Having participants complete the chart and fill in the missing labels of the fishhook diagram—eyelet, shank, belly, barb, tip, brings context to the session and provides a basis to talk about and understand the fishhook removal techniques (objective 2).

Diagrams of well-known methods of fishhook removal are provided and briefly discussed, and the remaining time is spent on self-directed hands-on learning. The methods of fishhook removal include the string technique, the push-through method, the needle (release) technique, and the cut it out technique. Participants attempt the different fishhook removal techniques excluding the cut it out technique on the injection pad trainer (objective 3).

Utilizing portable injection pad trainers requires no time to make or prepare a simulation model. Silicon gives the injection pad trainer a quality of skin resistance when removing the fishhook. Compared to previous simulations one benefit of the injection pad trainers is a potentially increased realism because of the ability to practice fishhook removal on different parts of the body. Injection pad trainers manufactured with a curved back allow them to fit securely on both distal extremities and the axilla. In addition to the main aspects of the fishhook removal techniques, participants learn patient positioning for maximum comfort and prevention of further injury. A dangling lure with multiple hooks on the foot or axilla hooks makes participants ensure their patient is not impaled a second time and that they readjust their patient during the removal of the fishhook. Participants learn strategies for and limitations of manipulating the hook, string, needle, and wire cutters. When cutting the hook participants learn to consider eye protection for themselves and their patient.

Durability of the pad allows for multiple attempts in performing each technique which is identified as valuable for improving the participants’ skill development. Injection pad trainers show signs of use, however, do not develop any significant tears that prevent them from being reused for multiple small group sessions.

### Recommended pre-reading for instructor

Review the provided handouts. Previously published educational materials on wound management for fishhook injuries and removal can be reviewed by instructors on UpToDate through searching Fish hook removal techniques.^6^ We also recommend Chapter 45: Soft Tissue Foreign Bodies of Tintinalli’s Emergency Medicine: A Comprehensive Study Guide, 9e and Chapter 26: Hunting and Fishing Injuries of Auerbach’s Wilderness Medicine, 7e.^7^,^8^ Tintinalli’s digital version has an accompanying instructional video performing fish hook removal on a cadaver. To note, while we feel the needle (release) techniques is an effective method with practice, in this video they do not recommend this method.^7^ Videos which demonstrate these various techniques on emergency department patients include those by Dr. Larry Mellick which can be found on YouTube.^9^

### Implementation Methods

This small group session may best be implemented as an accompaniment to educational workshops with multiples stations. For example, this fishhook removal session was one of six stations at a wilderness medicine training event for medical and nonmedical participants. It requires three minutes to set out the items needed for the simulation. Each rotation is effectively carried out with four to six participants and runs approximately thirty minutes before participants transition to another station.

### List of items required to replicate this innovation

1. Laminated tetanus prophylaxis chart, fishhook diagram, and fishhook removal pictures
2. Dry erase markers
3. Injection pad trainers with straps
4. Various fishhooks and lures
5. Fishing line or string
6. Wire cutters and hemostat/pliers
7. 18-Gauge needles
8. Safety glasses

### Approximate cost of items to create this innovation

The commercially available silicone sponge injection pad trainers used for this simulation are low cost. At the time of purchase of the materials for our simulation each injection pad trainer cost approximately $4.25 (when purchased as a set of eight). Twenty-five hooks of assorted sizes cost about $5. Other items including pliers, hemostats, and eye protection are often readily found in the clinical setting and frequently carried by fishers.

### Detailed methods to construct this innovation

**Figure 2:**
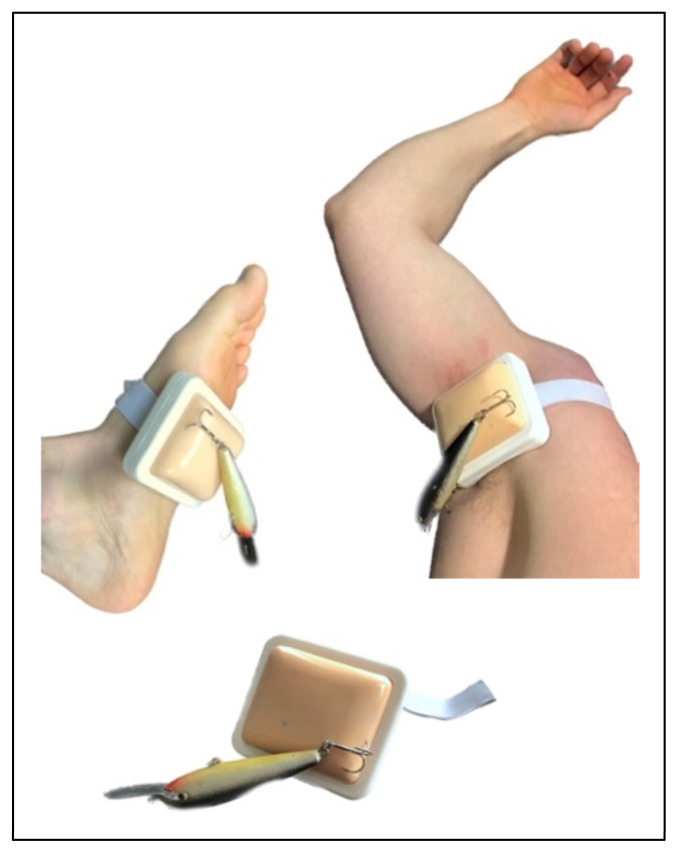
Commercially available silicone sponge injection pad trainers allow participants to practice fishhook removal techniques on various body parts and learn patient positioning.

1. Have a facilitator collect all items required for this simulation. Commercially available silicone sponge skin injection pad trainers can be purchased online. The Silicone Sponge Human Skin Injection Pad (Manufacturer VturboWay 8.4x7.5x3.8cm, tape size 2.5cm width × 50cm length, bottom pad thickness: 2cm, practice sponge thickness: 1.8cm) is one example (Figure 2).^5^ This injection pad trainer has straps that allow it to be secured with hook and loop closures to different extremities. Have one trainer for every two people. Print the included handouts on tetanus prophylaxis and fishhook diagram, as well as the instructions of the fishhook removal techniques. Laminate materials to allow for use with dry erase markers and increase durability.

### Tetanus prophylaxis chart and fishhook diagram

**Author’s own image** [Fig f1-jetem-8-4-i1]

2. To begin the simulation, instruct participants to do the following tasks. Choose one participant to serve as scribe for the group as they fill in the tetanus prophylaxis chart and fishhook diagram. Answers should be checked with the answer key.
3. To prepare to perform fishhook removal, participants should partner up, with one participant being the health care provider and the other the patient. They will place the pads on the feet, legs, axilla, arms, and hands, and then impale and remove hooks and lures. In addition to the manufacturer suggestion of wrapping the straps twice when placing on a distal extremity, a gently tied water knot (ring bend) adequately secures the pad when impaling or removing a fishhook. If a facilitator is present, they may choose to place the pad and hook themselves, mimicking a patient presenting with a hook already embedded.
4. Participants should review the fishhook removal instructions and attempt one method at a time. After attempting all the methods, they will change roles with their partner. As participants practice the various techniques, they can be guided to assist the patient in moving into an optimal position. A table or bench can be made available to participants to stabilize body parts. A variety of hooks including simple single tip hooks sizes #22 to #6 and treble hooks sizes #10 to 1/0, with and without lures, are provided to impale into the injection pad trainers. Fishing line can be used as string for the string method. 18-Gauge 1” needles are used for the needle technique. When practicing the push-through method and cutting hooks with wire cutters, participants must use the provided safety glasses. Practicing the cut it out technique should be avoided as it will quickly destroy the injection pad trainers. Throughout the session, dust and dirt stuck to the silicon are easily removed with rubbing alcohol and a microfiber cloth.

### Silicon sponge human skin injection pad trainer

**Author’s own image**

### Results and tips for successful implementation

To determine the effectiveness of this simulation it was implemented at an annual wilderness medicine medical student weekend training. Medical and physician assistant students from various medical schools in North Carolina, and their guests, were given surveys before starting and after completion of the simulation. Once collected, values of confidence and effectiveness in increasing skill-based competences that participants marked on a scale from low to high were measured with a ruler. The low end of the scale was given a value of 0% and high end was given a value of 100%. For participants who drew a circle on the line (n=12) the center of the circle was used to determine the percent value. A paired t-test was used to compare pre-simulation and post-simulation confidence. Histograms of survey answer distributions were binned up with intervals of 10. For perceived monetary value one participant responded with a range of values for which an average was taken.

Pre-activity and post-activity surveys were completed by 29/31 participants. As indicated by our survey results participants had some familiarity with fishing, fishhooks, and fishhook injuries, but not extensive knowledge or experience. 79% of participants had been fishing before (n=23). 14% of participants named two parts of a fishhook, however, no participants correctly named three parts. 24% correctly answered that a 3/0 hook is larger than a #3 hook. 17% of participants had prior experience with fishhook removal. 17% of participants knew one or more fishhook removal technique. The pre-activity mean confidence was 17% with a standard deviation of 19 and the post-activity mean confidence was 75% with a standard deviation of 15. The difference in mean confidence before and after the simulation was 58% (p<0.005). The mean level of participant perceived effectiveness of the simulation was 87% with a standard deviation of 14.[Fig f3-jetem-8-4-i1]

All participants who completed the survey responded that the simulation increased their understanding of when to use a particular fishhook removal technique over another or what technique they would attempt first. The simulation also helped all participants understand something previously not understood about fishhook removal. Furthermore, 100% of participants who completed the survey responded that based on the experience with the simulation, fishhook removal is a skill that healthcare providers should practice prior to performing on a patient.[Fig f4-jetem-8-4-i1]

For the push-through method, participants learned to invert the wire cutters when needed to cut the hook lower on the shank and gain a sense of the force needed to cut a variety of hooks. Initially participants commented on the difficulty of the needle method, however, they used this method in conjunction with the string technique. Through repeated attempts they learned that applying tension with the string allowed better visualization of the barb to both depress and disengage it with downward pressure and cover it with the needle. With practice, participants began to decrease tissue damage caused by large hooks by utilizing the needle to widen the entrance tract so they could more easily reverse the hook out of skin. It was observed that they also developed more proficiency in using the needle to release the small area of skin between the barb and rest of the hook. All these aspects likely factored into participants gaining increased confidence.

Based on our experience this simulation can be optimized for different teaching environments and skill levels. Lures that have treble hooks are typically more challenging to remove. As has been suggested in previous literature, participants can first cut the body of the lure or other parts of the treble hook away from the impaled hook to decrease the chance of secondary injury.^8^ Additionally, to increase the difficulty two of the barbs of the same treble hook can be inserted into the trainer or barbs of two different hooks on the same lure can be impaled. When considering eye protection for the push-through method, in addition to safety glasses and the use of hemostats to secure the tip, the hook can be covered with a cloth or bag prior to cutting.

### Pearls

1. Handouts on tetanus prophylaxis and the common parts of a fishhook with answer keys, along with instructions for fishhook removal methods, provide pre-learning for participants.
2. Silicon skin injection pad trainers can be secured to different body parts and impaled with various hooks in multiple configurations.
3. Students learn through observation of successful attempts and multiple repetitions of the string technique, the push-through method, the needle (release) technique for fishhook removal.

### Associated Content

- **Learner Materials**
  ○ Pre-activity Survey
  ○ Post-activity Survey
  ○ Tetanus Prophylaxis Chart
- **Instructor Materials**
  ○ Pre-activity Survey Answers
  ○ Tetanus Prophylaxis Chart Answers
  ○ Fishhook Removal Instructions

## Figures and Tables

**Figure f5-jetem-8-4-i1:**
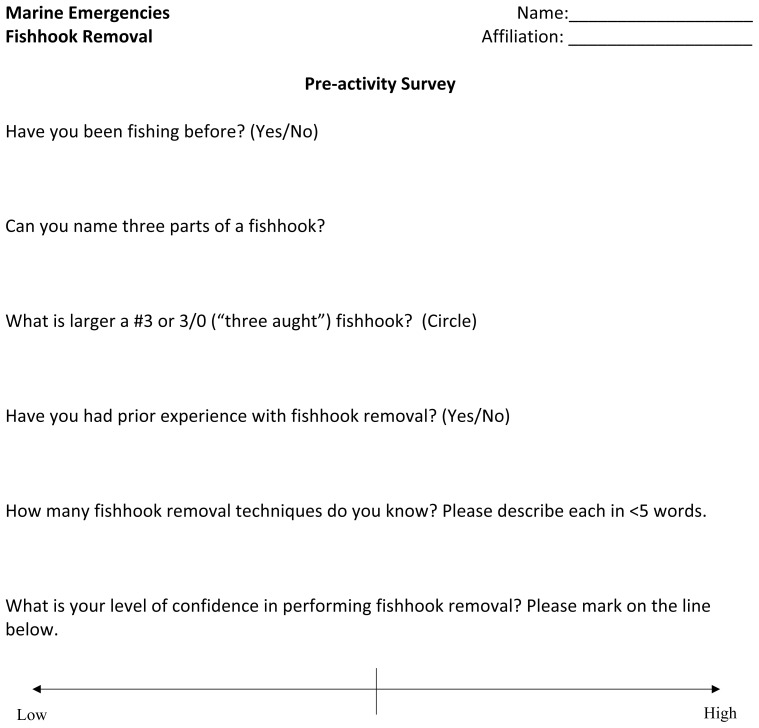


**Figure f6-jetem-8-4-i1:**
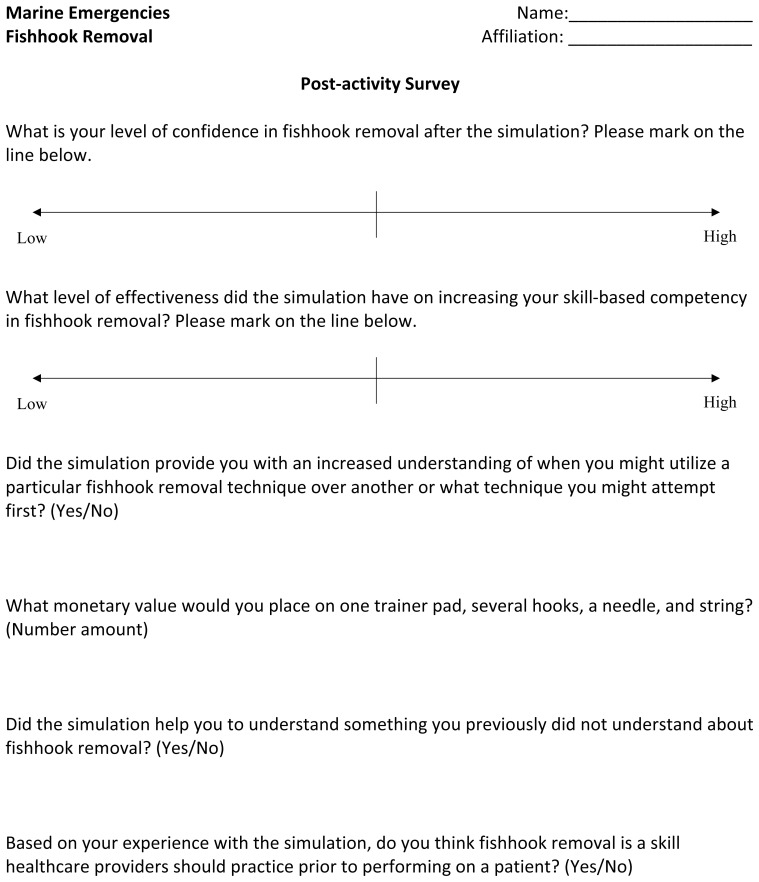


**Figure f7-jetem-8-4-i1:**
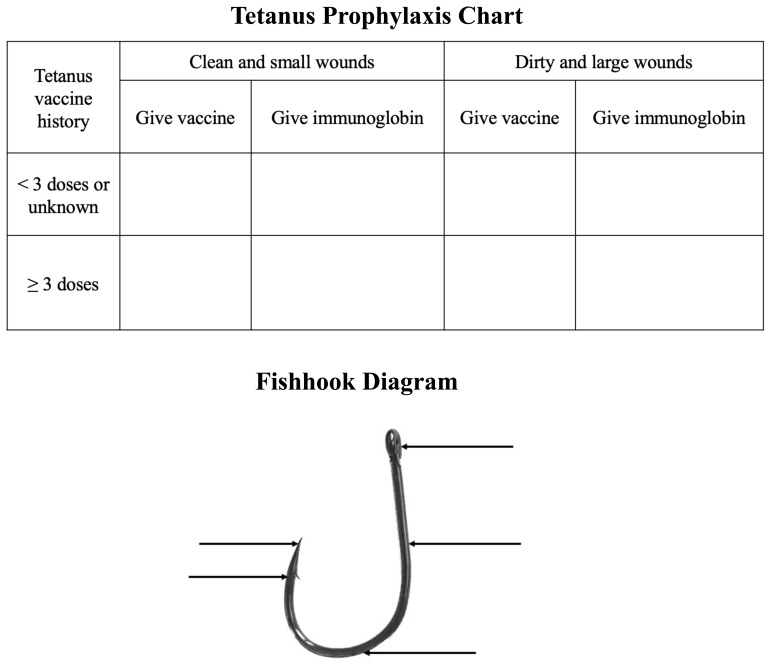


**Figure f8-jetem-8-4-i1:**
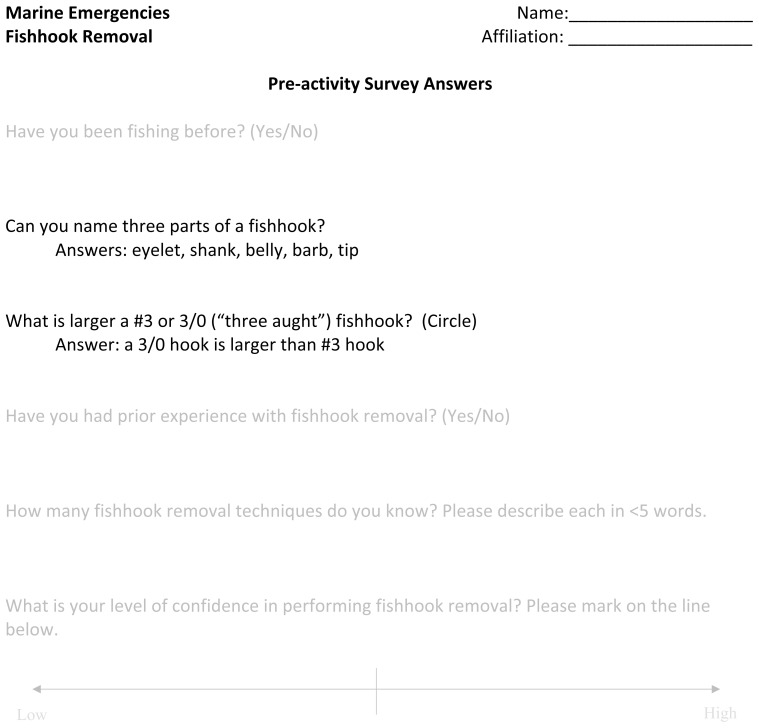


**Figure f9-jetem-8-4-i1:**
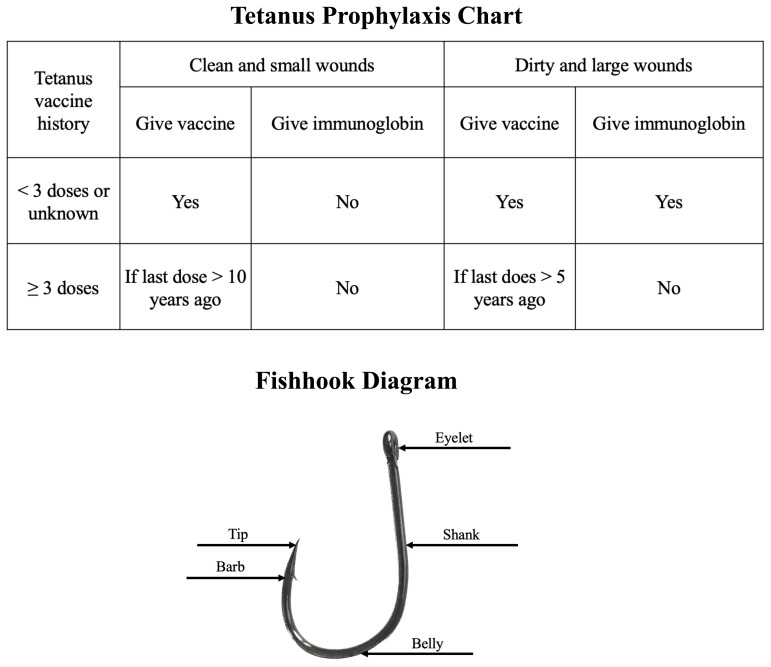


**Figure f10-jetem-8-4-i1:**
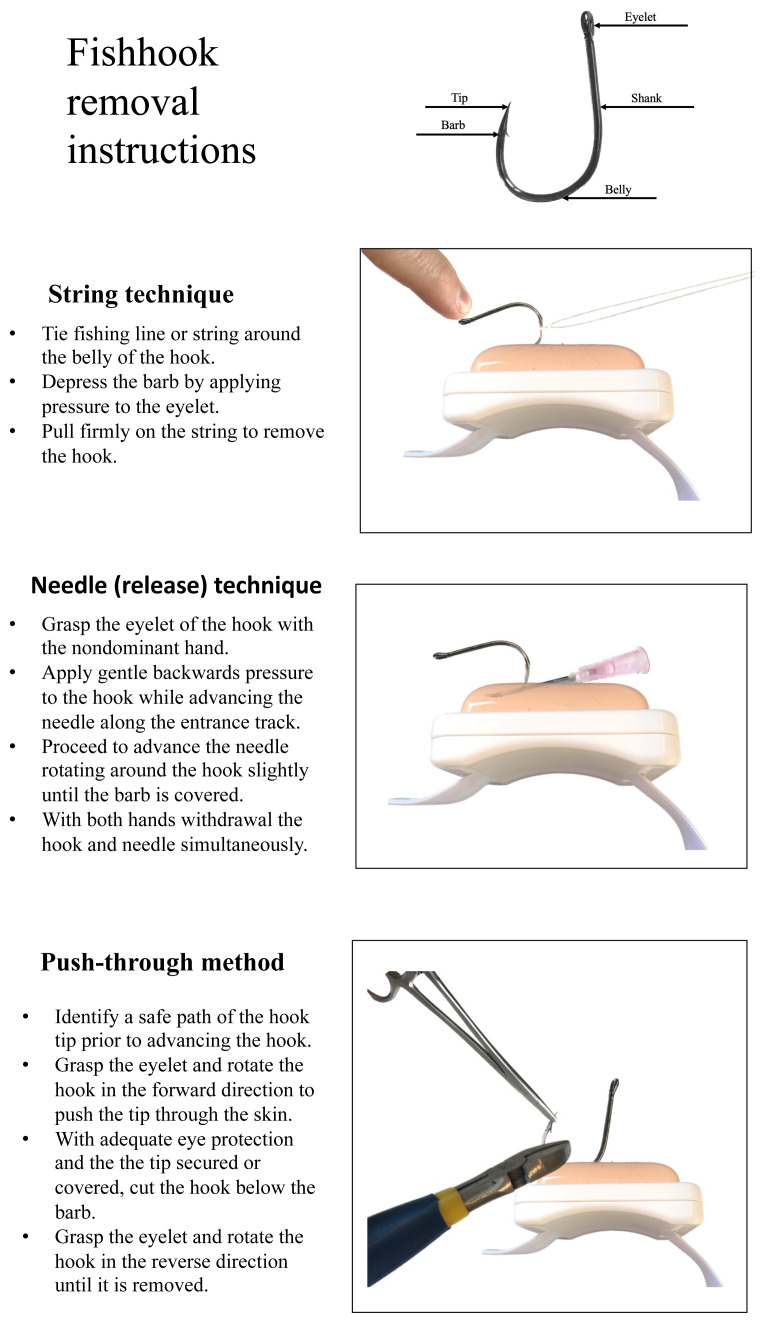


**Figure 1 f1-jetem-8-4-i1:**
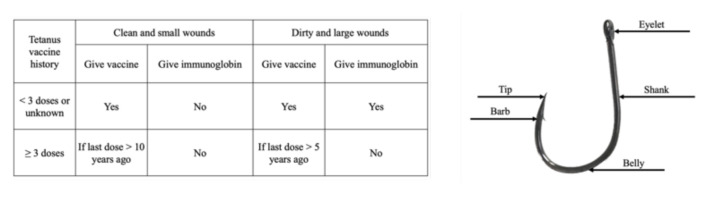
Tetanus prophylaxis chart and fishhook diagram bring added context to learning the fishhook removal techniques.

**Figure 3 f3-jetem-8-4-i1:**
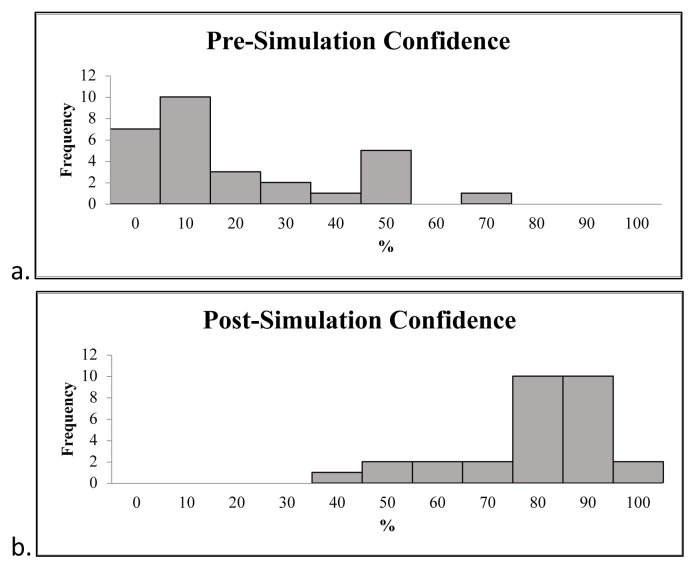
Histograms of pre-simulation confidence (a) and post-simulation confidence (b) show a significant increase.

**Figure 4 f4-jetem-8-4-i1:**
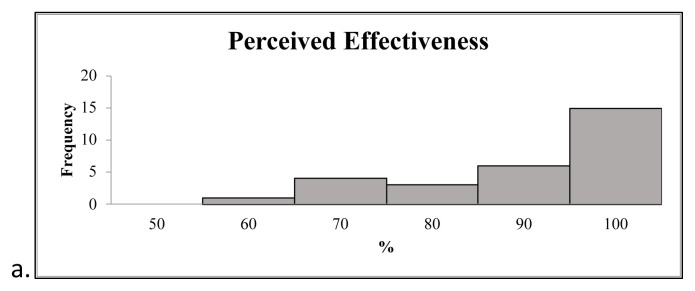

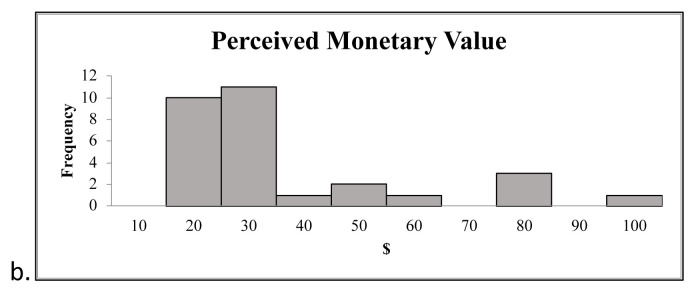
The distribution of participant perceived effectiveness of the simulation on increasing skill-based competency is high (a). Participant perceived monetary value of the simulation materials greater than actual cost (b).

## LEARNER MATERIALS

[Fig f5-jetem-8-4-i1] [Fig f6-jetem-8-4-i1] [Fig f7-jetem-8-4-i1]

## INSTRUCTOR MATERIALS

[Fig f8-jetem-8-4-i1] [Fig f9-jetem-8-4-i1] [Fig f10-jetem-8-4-i1]
